# Development and Evaluation of a Novel Cuproptosis-Related lncRNA Signature for Gastric Cancer Prognosis

**DOI:** 10.1155/2023/6354212

**Published:** 2023-02-11

**Authors:** Chunlin Yin, Ming Gao, Qi Wang, He Li

**Affiliations:** Department of Emergency, The Second Affiliated Hospital of Anhui Medical University, Hefei Anhui, China 230601

## Abstract

**Background:**

According to a growing body of research, long noncoding RNAs (lncRNAs) participate in the progress of gastric cancer (GC). Cuproptosis is a distinct kind of programmed cell death, separating it from several other forms of programmed cell death that may be caused by genetic programming. Consequently, it is crucial to examine cuproptosis-related lncRNAs (CRLs) prognostic importance for the prognosis and treatment response in GC.

**Method:**

The Cancer Genome Atlas (TCGA) database was used to retrieve RNA-seq data, pertinent clinical information, and somatic mutation data. A list of cuproptosis-related genes (CRGs) was obtained from prior work. We can distinguish prognostic CRLs using coexpression and univariate Cox analysis. Then, using CRLs, we developed a risk prediction model using multivariate Cox regression analysis and the least absolute shrinkage selection operator (LASSO) technique. To evaluate the diagnostic accuracy of this model, a Kaplan-Meier (K-M) survival analysis and a receiver operating characteristic (ROC) analysis were used. Moreover, the relationships between the risk model and immunological function, somatic mutation, and drug sensitivity were also investigated.

**Results:**

Using the multivariate Cox analysis technique, we developed a signature based on cuproptosis-related four lncRNAs. We then classified patients into high-risk and low-risk groups based on the likelihood of unfavorable outcomes. The model was subjected to further testing, including K-M survival analysis, ROC analysis, and multivariate Cox regression analysis, all of which proved the model's exceptional robustness and predictive capacity. In addition, a nomogram that has a strong capacity for prediction ability was built. This nomogram included age, gender, clinical grade, pathologic stage, T stage, and risk score. Furthermore, we discovered substantial disparities in immune function and the number of mutations carried by tumors between the high-risk and low-risk groups. Moreover, this research also found that the IC50 values for 27 chemotherapeutic drugs varied widely across patients within high- and low-risk groups.

**Conclusion:**

The proposed 4-CRLs signature is a promising biomarker to predict clinical outcomes in GC.

## 1. Introduction

Gastric cancer (GC) is one of the fatal gastrointestinal malignancies in the world and the third leading cause of cancer-related mortality. In 2020, a total of 1089103 new cases of stomach cancer and 768793 fatalities were attributed to *Helicobacter pylori* infection and poor lifestyles [1]. GC has brought a huge burden on Chinese residents [2]. Despite rapid advancements in surgical methods and adjuvant medicine, the overall survival rate (OS) of individuals with GC remains extremely low. The five-year survival rate is less than 20%, especially in persons with advanced GC [3]. The tumor node metastasis (TNM) classification is still a globally recognized standard for GC categorization. However, since each patient is unique and responds differently to treatment, the prognosis of people with the same TNM classification may be incorrect. As a consequence, improved prognostic markers for GC are urgently required.

Cuproptosis is a novel kind of cell death that occurs when copper is directly coupled with lipoylated components of the tricarboxylic acid (TCA) cycle. The cell is killed during this procedure. This process causes a buildup of lipoylated proteins, which leads to the loss of iron-sulfur cluster proteins, which leads to toxic protein stress and, eventually, cell death [4]. The molecular weight of long noncoding RNAs (lncRNAs) is more than 200 nucleotides [5]. Although they are not involved in protein translation, they play a crucial function in gene regulation. Many recent studies have shown that lncRNAs significantly regulate tumor proliferation, metastasis, invasion, and programmed death. For example, Lin et al. showed that lncRNA ITGB8-AS1 acts as a ceRNA to promote colorectal cancer growth and migration through integrin-mediated focal adhesion signaling [6]. One researcher recently found that lncRNA, namely, EIF3J-DT, could induce chemoresistance via autophagy activation in GC patients [7]. Additionally, the prognosis of GC can be predicted by detecting some lncRNAs in plasma or serum [8].

Nevertheless, the role of cuproptosis in cancer progression is unknown, and the role of cuproptosis-related lncRNAs (CRLs) in the prognosis of GC has also not been reported. In this study, we explored the role of CRLs in GC through TCGA database (http://portal.gdc.cancer.gov/). At the same time, the correlation between the expression of CRLs and the survival rate and clinicopathological parameters of GC patients was analyzed. Subsequently, we developed a predictive model based on CRLs and evaluated its ability to independently and accurately predict the prognosis of GC patients.

## 2. Methods and Materials

### 2.1. Flow Chart

### 2.2. Data Capture

**Figure 1:**
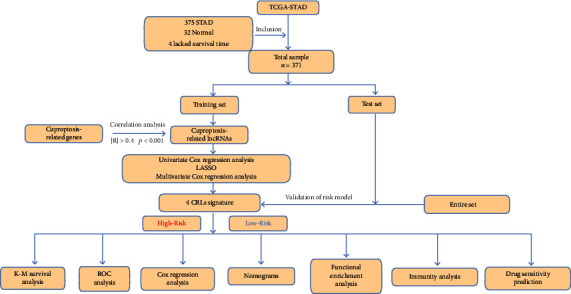
The flow chart of our study.

We acquired the stomach adenocarcinoma (STAD) data from TCGA database, including RNA sequencing data, pertinent clinical information, and somatic mutation data. TCGA database contains transcriptome data from 407 GC patients, including 375 STAD tissues and 32 normal tissues. In the interim, clinical data were also obtained. This data comprised gender, age, clinical stage and grade, and TNM stage. In addition, 19 cuproptosis-related genes (CRGs) were gathered from the previous study [4], and GENCODE v36 was applied for the annotation of the genes (https://www.gencodegenes.org/human/release36.html), (Figure 1).

### 2.3. Identification of Cuproptosis-Related lncRNAs

The list of CRGs was compiled from the prior literature, and Spearman's correlation coefficients were then computed based on CRGs and lncRNA expression patterns to identify CRLs (∣*R* | >0.4 and *p* < 0.001). In order to demonstrate the presence of a relationship between CRLs and the mRNAs to which they belong, a Sankey diagram was developed to depict the association between CRLs and their respective mRNAs.

### 2.4. Development of Cuproptosis-Related Risk Model

The candidate CRLs were first screened using univariate Cox regression analysis (*p* < 0.01) and the least absolute shrinkage selection operator (LASSO). For the generation and validation of risk models, 371 GC patients were randomly assigned to either a training cohort or a test cohort in a ratio of 6 : 4 (223 in the training cohort and 148 in the test cohort). In terms of clinical features, there was no discernible difference between the training and test cohorts (Table 1). Developing the prognostic risk signature of CRLs included using a linear combination of the expression values of all prognostic CRLs. This signature was then applied to the training set. The multivariable Cox proportional hazard regression analysis was used to assign weights to the predicted regression coefficients, as can be seen in Table 1: Risk score = (Expression of lncRNA_CDKN2B−AS1_ × 0.724957) + (Expression of lncRNA_VCAN−AS1_ × 0.666033) + (Expression of lncRNA_AL359704.2_ × 0.501099) + (Expression of lncRNA_HAGLR_ × 0.188734). Therefore, the median risk score was used to place each group of GC patients into either a high-risk or low-risk category.

### 2.5. Validation of Cuproptosis-Related Risk Model

We evaluated the predictive effectiveness of the risk model for overall survival (OS) and progression-free survival (PFS) using the Kaplan-Meier (K-M) survival analysis and receiver operating characteristic (ROC). Utilizing principal component analysis (PCA), the expression differential of CRLs in GC patients was determined. Using univariate and multivariate Cox proportional hazard regression, the independent prognostic determinants of OS were evaluated. Additionally, using the “rms” R package, nomograms were produced utilizing all independent prognostic markers and other clinical characteristics to analyze the 1-, 3-, and 5-year survival rates of GC patients. We also created calibration curves and assessed the consistency between nomogram-predicted and observed survival rates.

### 2.6. Functional Enrichment Analysis

The “limma” R package (∣log2 (fold change) | >1, FDR < 0.05) was used to identify the differentially expressed genes (DEG) between the high-risk group and the low-risk group, and the functional annotation was performed with “clusterprofiler” R package according to gene ontology (GO) and Kyoto Encyclopedia of Genes and Genomes (KEGG) (adjusted *p* value < 0.05).

### 2.7. Immunity Analysis and Drug Sensitivity Prediction

The two-sample Wilcoxon test was used to detect the difference in tumor mutation burden (TMB) between the high-risk and low-risk groups. The K-M survival analysis was used to evaluate the predictive ability of TMB for OS. Immune function analysis was done using the “GSVA” R package. The half-maximal inhibitory concentration (IC50) indicates the effectiveness of the substance in inhibiting specific biological or biochemical processes. The “pRRophetic” R package is applied to the IC50 of chemotherapy drugs.

### 2.8. Statistical Analysis

R.v.4.1.2 was used for statistical analyses, and the Chi-square test or the Wilcoxon test was used for differences analysis. The “survival” and “survminer” packages were used to conduct a Kaplan-Meier survival analysis. The “survivalROC” package is then used for ROC analysis. The AUC values were obtained to assess the prediction accuracy of the CRL-based prognostic model.

## 3. Results

### 3.1. Identification of Cuproptosis-Related lncRNAs in GC Patients

**Figure 2:**
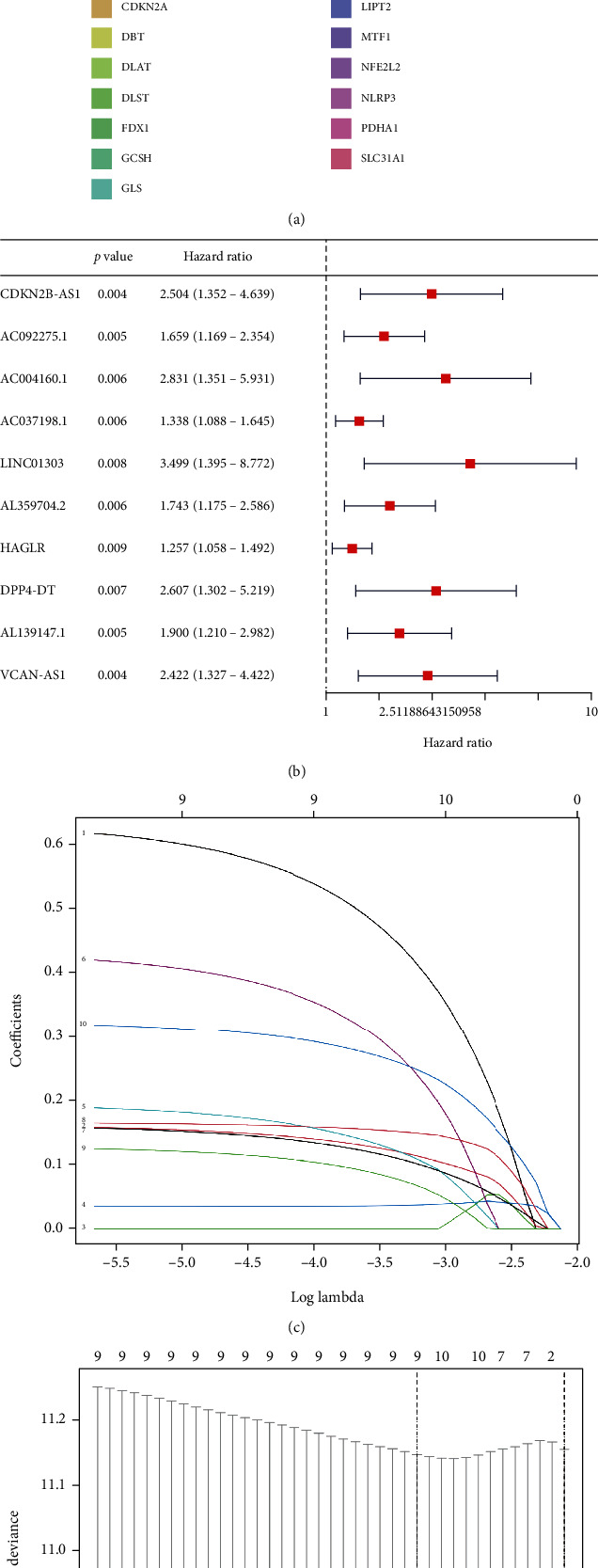
(a) The relationship between the novel lncRNA and CRGs. (b) Univariate Cox regression analysis. (c, d) LASSO regression analysis with minimum lambda value.

The cuproptosis-related genes (CRGs) were retrieved from the previous literature [4]. Subsequently, the expression of 19 CRGs and 16876 lncRNAs were downloaded from TCGA database. According to the filtering conditions (∣*R* | ≥0.4, *p* value < 0.001), a total of 430 CRLs were screened by Pearson's correlation analysis. A Sankey diagram also depicted the connection between CRLs and CRGs (Figure 2(a)).

### 3.2. Development of Risk Model Based on Cuproptosis-Related lncRNAs

Firstly, through univariate Cox regression analysis, 10 CRLs with significant correlation with OS in GC patients were screened via *p* value < 0.01 as the threshold (Figure 2(b)). Subsequently, to improve the prediction accuracy and avoid overfitting, a LASSO regression analysis was performed to screen out 9 CRLs with the optimal penalty parameter (*λ*) value (Figures 2(c) and 2(d)). Finally, a stepwise multivariate Cox proportional hazards regression analysis was performed, leading to identifying 4 lncRNAs (CDKN2B-AS1, VCAN-AS1, AL359704.2, and HAGLR) independently related to OS. Following this, a risk model was developed to predict the prognosis of GC patients.

### 3.3. Evaluation and Validation of the CRLs Prognostic Signature

**Figure 3:**
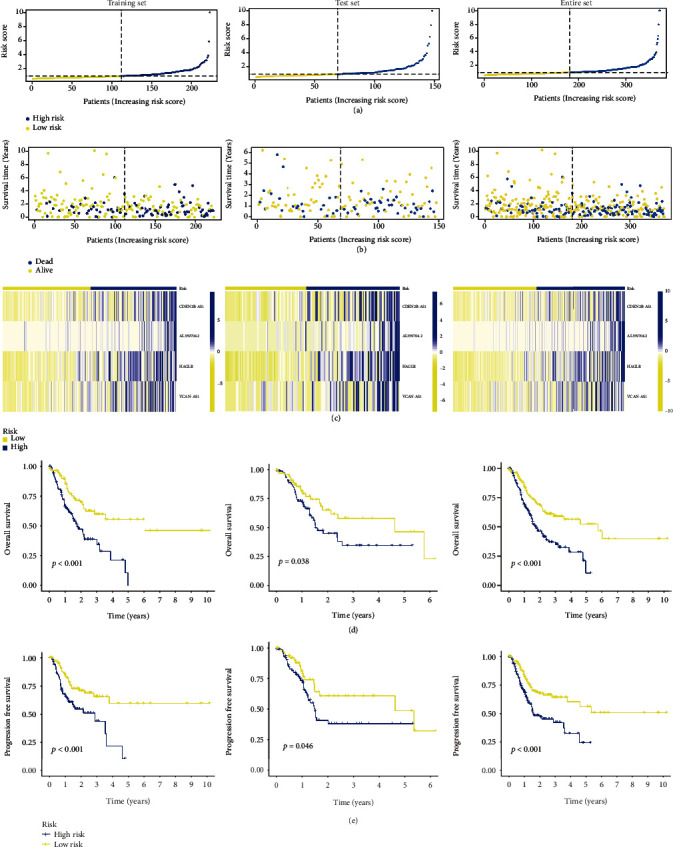
(a) Distribution of patients' risk scores. (b) OS status distribution. (c) Prognostic heatmaps. (d) K-M survival curve for OS. (e) K-M survival curve for PFS.

We classified the patients in the training set, test set, and entire set as high-risk or low-risk based on the median value of the risk score. We discovered that the sample distribution between high- and low-risk groups was appropriate based on the distribution of risk scores and OS status (Figures 3(a) and 3(b)). The heatmap in Figure 3(c) shows the expression of the 4 cuproptosis-related lncRNA signatures (CRLSig). Both the K-M technique and log-rank tests indicated the high-risk group have a poorer overall survival rate (OS) in training set (*p* < 0.001), test set (*p* = 0.038), and entire set (*p* < 0.001) (Figure 3(d)). We also addressed the effectiveness of the risk score in predicting progression-free survival (PFS). The K-M survival analysis also demonstrated that the risk score accurately predicted PFS in in training set (*p* < 0.001), test set (*p* = 0.046), and entire set (*p* < 0.001) (Figure 3(e)).

**Figure 4:**
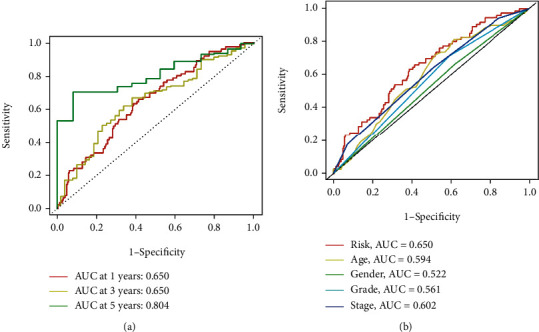
(a) ROC curve for the 1-, 3-, and 5-years. (b) ROC curve for the risk score and clinical characteristics.

**Figure 5:**
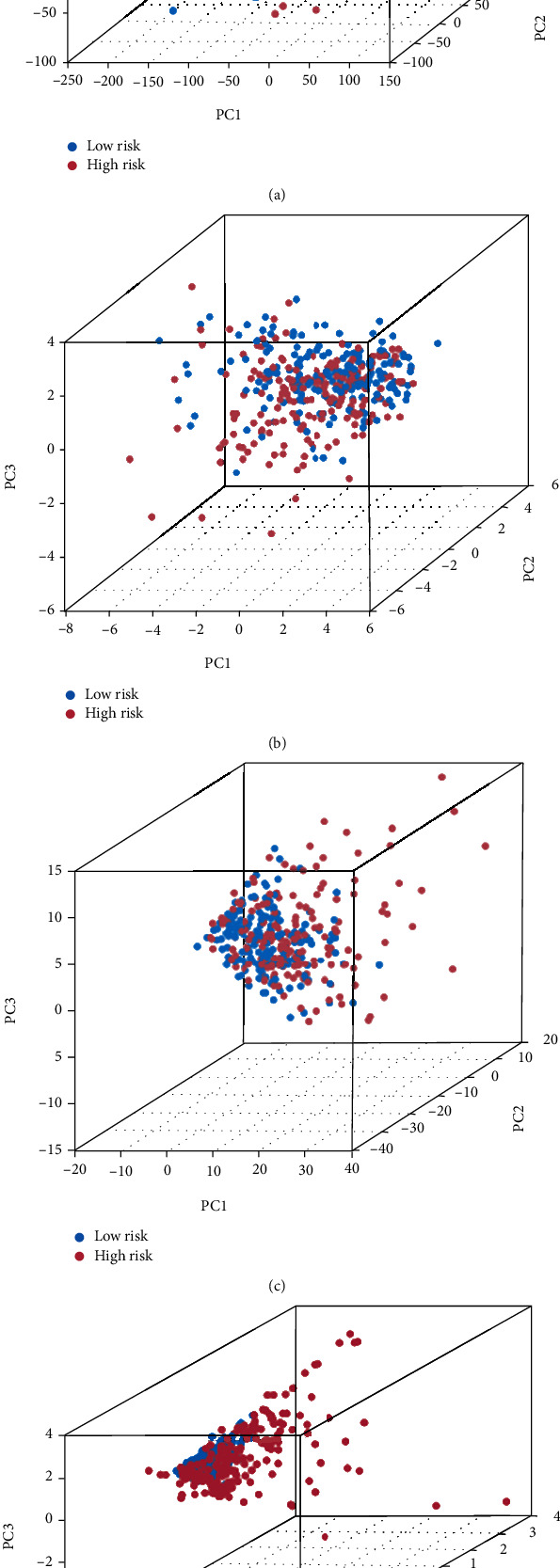
PCA based on (a) all genes, (b) cuproptosis-related genes, (c) cuproptosis-related lncRNAs, and (d) cuproptosis-related lncRNAs prognostic signature.

**Figure 6:**
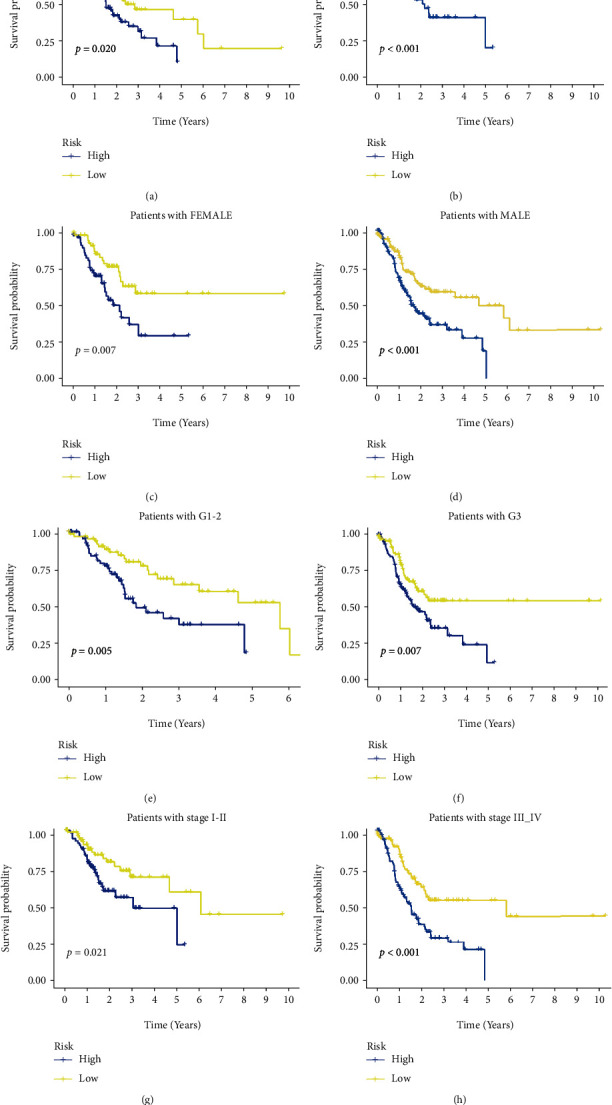
The OS of high-risk patients was significantly lower than that of low-risk patients in (a) age > 65 years, (b) age < 65 years, (c) female, (d) male, (e) G1 + G2, (f) G3, (g) stage I+II, and (h) stage III+IV.

All of the GC patients were subjected to the ROC analysis. The AUC of the ROC for the risk score was 0.650, 0.650, and 0.804 at 1, 3, and 5 years, respectively (Figure 4(a)). To test the novelty and sensitivity of the risk score in predicting the prognosis of GC patients, the AUC of the ROC curve for the risk score and other clinical parameters, such as age, gender, grade, and stages, was evaluated. It was corroborated by the findings that the AUC value of risk score was the highest among all variables (Figure 4(b)), which suggests that risk grade has great prediction effectiveness. In line with the above findings, the PCA analysis based on the model lncRNAs enables patients to be visually differentiated into two distinct groups (Figures 5(a)–5(d)). Besides, we also divided GC patients into subgroups according to their age, gender, disease progression stage, and sickness severity. The K-M survival analysis results showed that the OS of high-risk patients was significantly lower than that of low-risk patients in female group (*p* = 0.007), male group (*p* < 0.001), stage I-II group (*p* = 0.021), stage III-IV group (*p* < 0.001), etc. (Figures 6(a)–6(h)). Together, these results show that the 4 CRLSig would be a valuable GC prognostic model.

### 3.4. The Results of Univariate and Multivariate Cox Regression Analyses

**Figure 7:**
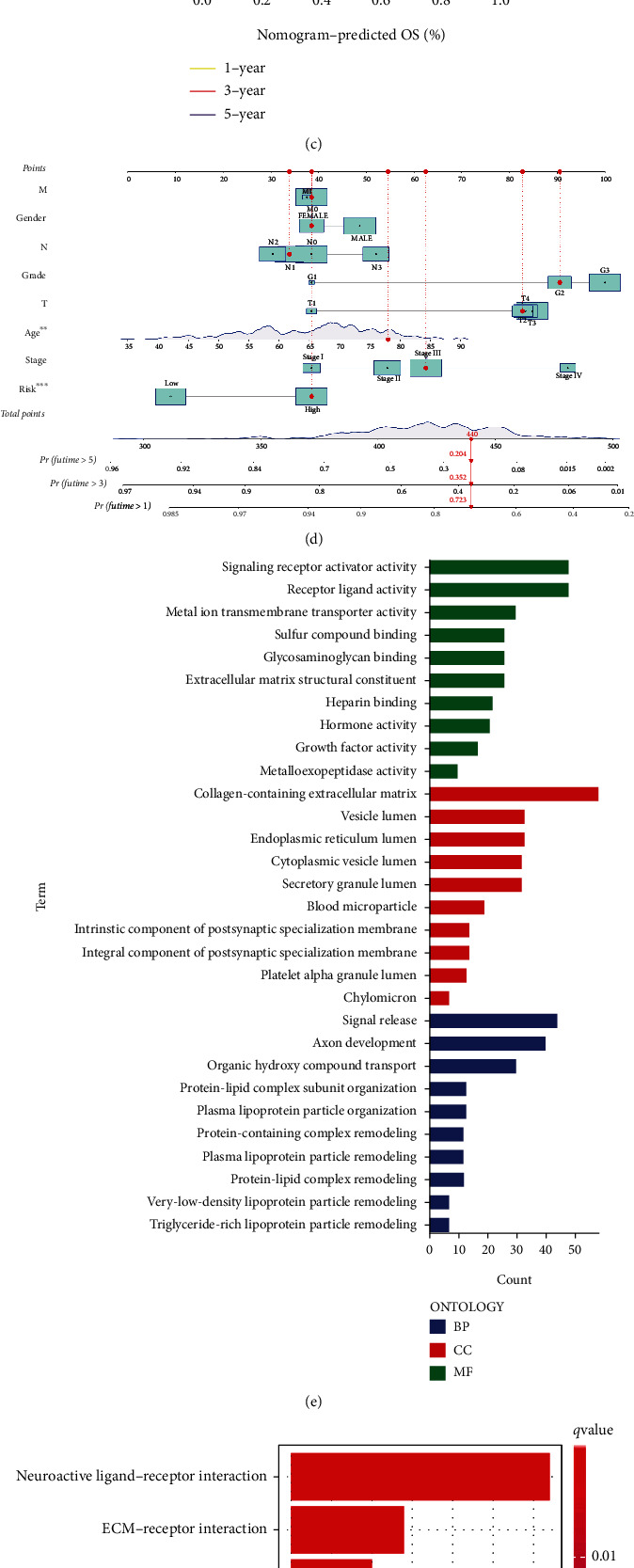
(a) Univariate Cox analysis. (b) Multivariate Cox analysis. (c) Calibration curves of the nomogram. (d) A nomogram was constructed to predict 1-, 3-, and 5-year survival rate of patient. (e) GO enrichment analysis. (f) KEGG enrichment analysis. (g) Immune function between two groups.^∗^*p* < 0.05; ^∗∗^*p* < 0.01; ^∗∗∗^*p* < 0.001.

We used univariate and multivariate Cox regression analysis to identify the independent factors that impact the prognosis of patients with GC. Age (*p* = 0.004, HR = 1.026), stage (*p* < 0.001, HR = 1.534), and risk score (*p* = 0.005, HR = 1.146) were independent variables that affected the prognosis of GC. Multivariate independent prognostic analysis showed that the age (*p* < 0.001, HR = 1.035), stage (*p* < 0.001, HR = 1.624), and risk score (*p* = 0.007, HR = 1.147) can be used as independent prognostic factors, which are high-risk factors (Figures 7(a) and 7(b)). Following this, in determining the potential clinical value of a predictive model based on 4 CRLSig, we created a nomogram that included risk scores in addition to other clinicopathological parameters to predict the 1-, 3-, and 5-year survival rate in patients who had GC. As can be seen in Figure 7(d), our findings indicate that a worse prognosis is associated with a higher estimated risk score. After that, a calibration curve was constructed to examine the degree to which the survival rate predicted by the nomogram was consistent with the observed survival rate. The findings demonstrate that the survival predictions for the next 1, 3, and 5 years are reasonably reliable (Figure 7(c)).

### 3.5. Functional Enrichment Analyses

In the differentially expressed genes (DEGs) found between the low-risk and high-risk groups, GO functional enrichment analysis and KEGG pathway enrichment analyses were carried out.  Figure 7(e) depicts the results of research involving GO enrichment. The primary functions of the enhanced biological process were signal release, axon development, and the transfer of organic hydroxy compounds. The majority of enhanced cellular components were found in the lumens of vesicles, endoplasmic reticulum, and cytoplasmic vesicles. Signaling receptor activator activity, receptor-ligand activity, and sulfur compound binding were the primary molecular functions. According to the findings of the KEGG analysis, many pathways connected to digestion had been considerably enriched, including protein digestion and absorption pathway, fat digestion and absorption pathway, and vitamin digestion and absorption pathway (Figure 7(f)).

### 3.6. Cancer-Related Gene Mutation between Two Groups

**Figure 8:**
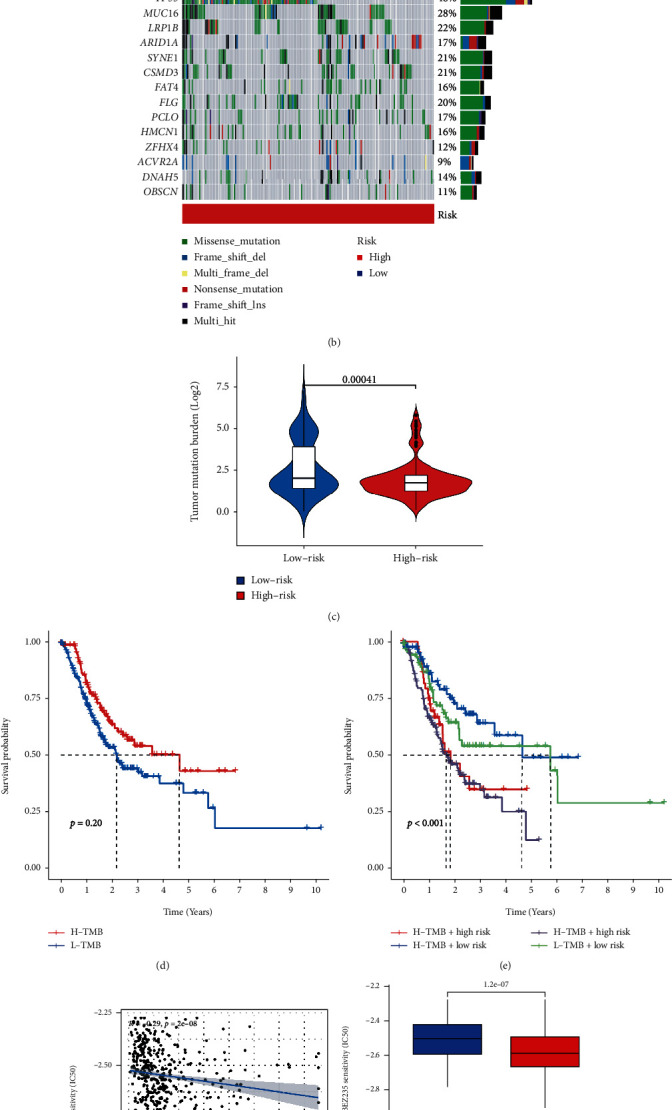
(a. b) Waterfall plot displaying the mutation profile of genes with high mutation rates in the low- and high-risk groups. (c) The difference in TMB between the low- and high-risk groups. (f–i) Drug resistance and sensitivity analysis between two groups.

With the use of the “maftool” package, a comparison was made between the distribution differences of somatic mutations found in groups with high and low-risk scores. The waterfall diagram shows that the low-risk group has a greater gene mutation rate than the high-risk group (Figures 8(a) and 8(b)). Besides, the low-risk group's tumor mutation burden (TMB) was significantly higher than the high-risk group in the TMB quantification analysis (*p* < 0.001) (Figure 8(c)). Based on the TMB cutoff value provided by the ‘survminer' package, all of the GC patients in our research were classified as either having a low or high TMB level. The K-M method and log-rank tests illustrate that patients in the TMB^high^ group (*p* = 0.02) and risk^low^ + TMB^high^ group (*p* < 0.001) had better OS than other groups (Figures 8(d) and 8(e)).

### 3.7. Immunity Analyses and Drug Sensitivity between Two Groups

We quantified the infiltrating scores of immunity-related activities in two groups to get a deeper understanding of the connection between the prognosis of GC and the immunological condition of the patient. Immune function scores exhibited substantial variations, including Type_II_IFN_Respons, APC_co_inhibition, T_cell_co_inhibition, Cytolytic_activity, inflammation-promoting, HLA, and MHC_ class_I between the low-risk and high-risk groups (Figure 7(g)). A Spearman's correlation analysis was conducted to investigate how the risk score impacts medication response to evaluate the relationship between the risk score and the IC50 for various pharmaceuticals. Twenty-seven drugs were connected with risk scores. In regards to risk scores, nine of these drugs demonstrated drug sensitivity, including PI3K/mTOR signaling inhibitor BEZ235 (Cor: 0.29, *p* < 0.001, Figures 8(f) and 8(g)), and 18 drugs exhibited drug resistance related to the risk score, such as Mitomycin (Cor: 0.27, *p* < 0.001, Figures 8(h) and 8(i)).

## 4. Discussion

Many scheduled and precisely controlled programmed cell death during the development of multicellular organisms, such as apoptosis, necroptosis, pyroptosis, and ferroptosis. Among them, ferroptosis is a new cell death mode named in 2012 [9], which plays a regulatory role in the occurrence and development of various tumors [10]. Like iron, copper is also an indispensable trace element in all organisms and usually maintains very low levels in mammalian cells. When the concentration of a copper ion in cells exceeds the threshold of maintaining a steady-state mechanism, it will also show cytotoxicity and lead to cell death [11]. Recent research conducted by Tsvetkov et al. conclusively showed that copper-dependent death is caused by the direct connection of copper with the lipoylated component of the tricarboxylic acid (TCA) cycle. It leads to the aggregation of lipoylated proteins and the subsequent loss of iron-sulfur cluster proteins, which leads to proteotoxic stress and, ultimately, cell death. This copper-dependent cell death was defined as cuproptosis [4].

Many noncoding genes have been discovered in recent years due to the advancement of high-throughput sequencing technology, and they play a critical role in the occurrence and progression of GC. According to a significant amount of past research [12–14], lncRNAs are involved in various biological processes, including GC's development, invasion, and metastasis. There is currently no study on how cuproptosis plays a part in the pathophysiological process of GC since it is a novel cell death method. There are no publications on the association between CRLs and the prognosis of GC patients. In this research, we proposed that CRLs may effectively indicate GC prognosis. We investigated the connection between the expression of CRLs and the survival or clinicopathological parameters of patients with GC using TCGA database. In addition, we developed a prognostic signature based on 4 CRLsig and tested its capacity to predict the prognosis of GC patients independently and effectively. This research developed a risk model based on 4 CRLsig to predict OS in GC patients.

First, 430 CRLs were obtained using coexpression analysis. Then GC patients were randomized into training and test groups (ratio of 6 : 4). Prognostic CRLs were defined using the LASSO regression and Cox regression model. The patients were split into high-risk and low-risk, based on their risk scores. It was noted that significant differences in OS existed between the two groups. The AUC also verifies the prediction ability of CRLsig. The Cox regressive analysis further verified the risk score was an independent prognostic factor for GC. The PCA analysis intuitively identified high- and low-risk groups. In addition, this model was also validated in the test and the entire group. Lastly, we identified 4 CRLsig, including VCAN-AS1, HAGLR, CDKN2B-AS1, and AL359704.2. One recent study reported that VCAN-AS1 could downregulate the expression of TP53 and promote the progress of GC by interacting with eIF4A3 while silencing VCAN-AS1 could inhibit cell proliferation, migration, and invasion but enhance apoptosis [15]. Meanwhile, HAGLR is highly expressed in GC tissues and cells and was found as a molecular sponge of miR-338-3p to promote 5-Fu resistance in GC via targeting the LDHA-glycolysis pathway [16]. Furthermore, voluminous literature has manifested that VCAN-AS1 and HAGLR also play a regulatory role in the occurrence and development of various tumors except for GC [17–20]. To our knowledge, CDKN2B-AS1 is abnormally expressed in various tumors [21–23]. However, CDKN2B-AS1 has not been reported in GC, which means that our results indicate that further research is necessary. Besides, we have not yet found studies on the significance of AL359704.2 in GC or other tumors. Our results show for the first time that these two lncRNAs are related to the prognosis of GC, and their potential mechanisms in GC need to be further explored.

Additionally, we integrated risk scores and predictive clinical features (including age, sex, pathological stage, grade, and T stage) to construct a nomogram for predicting the prognosis of patients. The results displayed that the higher the calculated risk score, the worse the predicted prognosis, which indicates that the nomogram provides a personalized and accurate survival prediction. To better understand the interaction between DEGs, we further performed a functional enrichment analysis. The KEGG pathway enrichment analysis reveals that DEGs were mainly involved in several digestion-related pathways such as protein digestion and absorption, fat digestion and absorption, and vitamin digestion and absorption. All functions were closely related to digestion and absorption, which may show that changes in the digestive function of the stomach and eating habits may have close links with the changes from healthy tissue to GS.

As a biomarker, TMB can predict immunotherapy's efficacy for various tumors. The higher the TMB, the more new antigens can be recognized by T cells, and the better the effect of immunotherapy [24, 25]. To further explore the role of TMB in the prognosis of GC, we divided the patients with GC into the high TMB group and low TMB group. The K-M analysis revealed that higher TMB was associated with better survival outcomes. Currently, chemotherapy is still an important method for treating GC, especially advanced GC [26], and drug resistance is the main cause of treatment failure [27]. Therefore, we analyzed the resistance and sensitivity of chemotherapeutic drugs to validate the predictive ability of CRLsig in determining treatment effectiveness. Our results presented that the high-risk group had a good response to BEZ235, an inhibitor of PI3K/mTOR signaling, while mitomycin could bring more benefits to the low-risk group. All of the findings above indicate that risk score-based classification has the potential to guide individualized chemotherapy and immunotherapy treatment strategies for individual tumors, hence improving the prognosis of patients with GC.

There are a few limits to our study despite our best efforts. Firstly, cuproptosis, a newly identified kind of cell death, has an unknown role in tumor growth. Secondly, it is necessary to do further in-depth research on the links between cuproptosis and CRLs since the precise mechanism linking the two processes is unknown. Thirdly, we only performed internal validation of the model through TCGA database and did not find a suitable external database to evaluate the model performance further. Therefore, in the future, we plan to conduct a retrospective analysis of previous gastric cancer data and evaluate its clinical application through our data.

To summarize, cuproptosis is a newly discovered kind of programmed cell death. Our findings could provide new insights into the molecular mechanisms involved in the genesis and progression of GC.

## Figures and Tables

**Table 1:** The clinical features between the training and test cohorts.

Covariates	Type	Total	Test	Train	*p* value
Age	<=65	163 (43.94%)	70 (47.3%)	93 (41.7%)	0.3471
	>65	205 (55.26%)	77 (52.03%)	128 (57.4%)	
	Unknown	3 (0.81%)	1 (0.68%)	2 (0.9%)	

Gender	Female	133 (35.85%)	58 (39.19%)	75 (33.63%)	0.3259
	Male	238 (64.15%)	90 (60.81%)	148 (66.37%)	

Grade	G1	10 (2.7%)	5 (3.38%)	5 (2.24%)	0.7846
	G2	134 (36.12%)	53 (35.81%)	81 (36.32%)	
	G3	218 (58.76%)	85 (57.43%)	133 (59.64%)	
	Unknown	9 (2.43%)	5 (3.38%)	4 (1.79%)	

Stage	Stage I	50 (13.48%)	17 (11.49%)	33 (14.8%)	0.7499
	Stage II	111 (29.92%)	42 (28.38%)	69 (30.94%)	
	Stage III	149 (40.16%)	60 (40.54%)	89 (39.91%)	
	Stage IV	38 (10.24%)	17 (11.49%)	21 (9.42%)	
	Unknown	23 (6.2%)	12 (8.11%)	11 (4.93%)	

T	T1	18 (4.85%)	8 (5.41%)	10 (4.48%)	0.9247
	T2	78 (21.02%)	31 (20.95%)	47 (21.08%)	
	T3	167 (45.01%)	67 (45.27%)	100 (44.84%)	
	T4	100 (26.95%)	37 (25%)	63 (28.25%)	
	Unknown	8 (2.16%)	5 (3.38%)	3 (1.35%)	

M	M0	328 (88.41%)	129 (87.16%)	199 (89.24%)	0.3012
	M1	25 (6.74%)	13 (8.78%)	12 (5.38%)	
	Unknown	18 (4.85%)	6 (4.05%)	12 (5.38%)	

N	N0	108 (29.11%)	36 (24.32%)	72 (32.29%)	0.4562
	N1	97 (26.15%)	39 (26.35%)	58 (26.01%)	
	N2	74 (19.95%)	32 (21.62%)	42 (18.83%)	
	N3	74 (19.95%)	32 (21.62%)	42 (18.83%)	
	Unknown	18 (4.85%)	9 (6.08%)	9 (4.04%)	

## Data Availability

The data used to support the findings of this study are available from the corresponding author upon request.
